# Physical activity and mental health: Does physical disability affect their association?

**DOI:** 10.1177/13591053251404136

**Published:** 2026-01-06

**Authors:** Emma M. Connell, Janine V. Olthuis

**Affiliations:** 1 University of New Brunswick, Fredericton, NB, Canada

**Keywords:** anxiety, anxiety sensitivity, depression, physical activity, disability

## Abstract

Though the positive association between physical activity (PA) and mental health is well established, this relationship is mixed among those with physical disabilities (PDs). Exploration of potential mediating (e.g. anxiety sensitivity; AS) and moderating (e.g. disability status) factors is needed to understand these inconsistencies. This study investigated (a) the moderating role of PD on the association between PA and anxiety and depression symptoms and (b) the mediating role of AS in this association. Participants (*N* = 231) who identified as someone with an acquired disability, a congenital disability, or no disability completed a survey including measures of PA, AS, anxiety, and depression. Results showed a significant moderating role for disability. Furthermore, there were significant differences between disability groups in the mediating role of AS in this association. The results emphasize the complex interplay between PA and anxiety and depression and how the association may be impacted by PD.

## Introduction

Individuals with physical disabilities (PDs; e.g. spinal cord injury (SCI), multiple sclerosis (MS), muscular dystrophy (MD), cerebral palsy, rheumatoid arthritis, post-polio syndrome) experience a wide range of challenging mental health outcomes. Many studies have found high rates of anxiety and depression in people with PDs, seemingly higher than in non-disabled samples, though there are few direct comparisons of these groups (Boeschoten et al., 2017; Lim et al., 2017; Sareen et al., 2005). Research is needed to identify ways to improve mental health in individuals with PDs. In populations without PDs, increased physical activity (PA) is associated with lower anxiety and depression, and has even been shown to effectively treat clinical anxiety and depression (Rebar et al., 2015; Wedekind et al., 2010; Weinstein et al., 2017; Wipfli et al., 2008). Typically, research suggests that only moderate-to-vigorous intensity PA is associated with anxiety and depression (Schuch et al., 2016; Wipfli et al., 2008). Nevertheless, some studies have also found that lighter intensity PA may also be associated with lower anxiety and depressive symptoms (Merom et al., 2008; Singh et al., 2005) and may be enjoyed more than vigorous PA (Teychenne et al., 2020).

There is much less research on the anxiolytic and anti-depressant effects of PA in those with PDs and existing studies have conflicting findings. One longitudinal study (*N* = 1218; Battalio et al., 2020) that included people with PDs found that moderate-to-vigorous, but not mild, PA was associated with a decrease in anxiety and depressive symptoms. Similarly, Vita et al. (2020) found that individuals with neuromuscular diseases (e.g. MD) who played sports had lower depressive symptoms than those who did not play. Conversely, cross-sectional studies of individuals with a SCI have found no association between PA and anxiety and depression or a significant negative association between mild, but not moderate or vigorous, PA and anxiety and depression (Bombardier et al., 2012; Connell and Olthuis, 2023; Tawashy et al., 2009). Further research is needed to clarify the PA–mental health association among those with a PD. This is particularly true as those with PDs are much less physically active than those without a disability (Jung et al., 2018; Martin Ginis and Hicks, 2007); barriers to PA for those with disabilities likely contribute to this discrepancy (Rimmer et al., 2004; Wilcox et al., 2006).

Further elucidation of the relation between PA and mental health among those with PDs may come from an investigation of the potential mechanisms in this association. One potential mechanism is anxiety sensitivity (AS). AS is a fear of arousal-related sensations (e.g. increased heart rate) that arises due to beliefs that these sensations will lead to physical, cognitive, or social catastrophe (Reiss and McNally, 1985). Though AS seems similar to trait anxiety, trait anxiety involves a fearful response to general stressors whereas AS involves a fearful response to the anxiety-related symptoms (Taylor et al., 1991). Research with individuals without a PD has shown that individuals with high versus low AS are more likely to avoid PA (McWilliams and Asmundson, 2001; Moshier et al., 2016). However, research also shows that PA can reduce AS (Jacquart et al., 2019). In fact, AS mediates the relation between PA and anxiety and depression. For example, Broman-Fulks et al. (2018) found that reductions in AS following PA accounted for 40% of the anxiolytic and anti-depressant effects of PA in a community sample.

Given the centrality of how one experiences and interprets physiological sensations to AS and the fact that individuals with PDs may experience many physical sensations differently than those without disabilities (e.g. numbness or a decrease in sensation, chronic pain, disruptions to thermoregulation and heart rate), the relation between PA and AS (and therefore between PA and anxiety and depression) may be disrupted (Broman-Fulks et al., 2018; Forsyth et al., 2019; Kesselring, 2003). In fact, a recent study of individuals with a SCI (Connell and Olthuis, 2023) found that AS was not associated with PA among those with paraplegia but was positively associated with PA among those with tetraplegia. The shift in the directionality of the relation (i.e. different than the negative association between AS and PA found in those without disabilities) may be due to the changes that occur to the experience and interpretation of physiological sensations after a SCI. Specifically, the authors posit that though individuals with tetraplegia may have high AS, they may not experience the sensations associated with AS when they do PA and therefore they may not avoid PA in the same way as other samples with high AS (Connell and Olthuis, 2023). Importantly, this is the only study that has considered the role of AS in populations with PDs. More research is needed to replicate and extend this finding.

Importantly, people with PDs are a diverse group with varying experiences. One important distinction is between those who have a congenital disability (they were born with their disability), and those who acquire their disability later in life. When it comes to PA and mental health, research comparing these groups is limited. Some research suggests they do not differ in PA participation or athletic identity (Cho et al., 2021; Rougeau et al., 2025). On the other hand, research has found higher rates of depression among those with an acquired versus a congenital disability (Kim and Park, 2024). This may be because individuals with an acquired disability feel a greater sense of loss after their diagnosis, have more difficulty accepting their disability identity, and have internalized society’s prejudices about disability more than those with a congenital disability (Bogart, 2014; Bogart et al., 2012; Jama et al., 2021). The distinction between congenital and acquired disability may also have implications for how one experiences and interprets physiological arousal. Those with an acquired disability experience a *change* in their physiology, whereas individuals with a congenital disability may experience some physiological sensations that differ from those without a disability but have had this lived experience for most, if not all, of their lives. This difference between acquired and congenital disabilities may have important implications for AS and how it relates to PA and anxiety and depression, but this has not yet been explored.

### Study aims

The goal of this study was to better understand the associations between PA and anxiety and depression in people with PDs. Specifically, we investigated (aim 1) whether disability status (i.e. acquired, congenital, or no disability) moderated the relation between PA and anxiety and depression. We hypothesized that PA would be negatively associated with anxiety and depression for those with no disability (as in prior research; Wedekind et al., 2010) and those with a congenital disability (as among those with neuromuscular disorders; Vita et al., 2020). For those with an acquired disability, however, we hypothesized that there would be no association between PA and anxiety and depression (as among those with a SCI; Bombardier et al., 2012; Connell and Olthuis, 2023; Tawashy et al., 2009).

Next, to explore potential mechanisms in the association between PA and anxiety and depression, we investigated (aim 2) whether disability status moderated the relation between PA and AS. We hypothesized that PA and AS would be negatively associated amongst those without a disability (as in prior research; DeWolfe et al., 2023) but that the effect of PA on AS would be weaker amongst those with an acquired or congenital PD (as in prior research; Connell and Olthuis, 2023). We then investigated (aim 3) whether AS mediated the association between PA and anxiety and depression and whether disability status moderated this mediation. We hypothesized that moderated mediation would emerge, such that AS would partially mediate the association between PA and anxiety and depression (i.e. less PA would be associated with higher AS which in turn would be associated with more anxiety and depressive symptoms; Broman-Fulks et al., 2018) but only among people without disabilities.

## Method

### Participants

Participants were adults (⩾19 years of age) recruited from Canada and the United States. A total of 231 participants (Table 1) were included in the analysis including 41.6% (*n* = 96) with no disability, 30.3% (*n* = 70) with a self-identified acquired disability (arthritis, MS, SCI, etc.), and 28.1% (*n* = 65) with a self-identified congenital disability (scoliosis, MD, cerebral palsy, etc.). Those in the disability groups reported a wide variety of diagnoses with the most common being arthritis (various types, *n* = 20), Fibromyalgia (*n* = 7), scoliosis (*n* = 7), Ehlers Danlos Syndrome (*n* = 6), and MS (*n* = 5). For those with an acquired disability, the mean time since diagnosis was 10.29 years (SD = 11.87) and time since diagnosis was significantly correlated with depression (*r* = −0.25, *p* = 0.045) but not anxiety, AS, or PA. Most of the sample identified as White (73.2%), married or in a common law relationship (38.5%), university-educated (43.7%), and earning $85,000 a year or less (64.5%). Of those who had a disability, 52.2% reported that they did not use a mobility aid. Approximately half of the sample (52.0%) reported having been diagnosed with a psychological disorder, most commonly an anxiety disorder (39.5%) and/or depression (34.5%).

**Table 1.** Demographics and descriptive statistics.

Demographic variables	No disability *n* = 96	Acquired disability *n* = 70	Congenital disability *n* = 65	Total sample *N* = 231
Gender				
Woman	56.3%	60.0%	52.3%	56.3%
Man	42.7%	32.9%	40.0%	39.0%
Nonbinary or Genderqueer	1.0%	5.7%	6.2%	3.9%
Marital status				
Single	28.1%	30.0%	47.7%	34.2%
Relationship (<6 months)	6.3%	1.4%	4.6%	4.3%
Relationship (>6 months)	15.6%	17.1%	24.6%	18.6%
Married	49.0%	42.9%	18.5%	38.5%
Divorced	1.0%	8.6%	4.6%	4.3%
Income				
<$35,000	11.5%	31.4%	33.8%	23.8%
$35,001−$60,000	12.5%	18.6%	33.9%	20.4%
$60,001−$85,000	20.8%	24.3%	15.4%	20.4%
$85,001–$110,000	22.9%	10.0%	4.6%	13.9%
$110,001–$135,000	10.4%	7.1%	1.5%	6.9%
>$135,001	17.7%)	1.4%	4.6%	10.8%
Education				
Some high school	0.0%	0.0%	1.5%	0.4%
High school (or GED)	10.4%	25.7%	32.3%	21.2%
Trade school	7.3%	10.0%	7.7%	8.2%
Community college	9.4%	20.0%	9.2%	12.6%
University	53.1%	37.1%	36.9%	43.7%
Post-graduate studies	15.6%	5.7%	12.3%	11.7%
Ethnicity				
Indigenous	1.0%	2.9%	0.0%	1.3%
Caucasian	69.8%	75.7%	75.4%	73.2%
Hispanic	5.2%	5.7%	9.2%	6.5%
Black	3.1%	2.9%	3.1%	3.0%
Asian	17.7%	7.1%	1.5%	10.0%
Mixed	2.1%	5.7%	6.2%	4.3%
Other	0.0%	0.0%	1.5%	0.4%
Psych DX	29.2%	65.7%	70.8%	52.0%
Age	34.51 (10.80)	42.27 (14.3)	38.66 (14.31)	38.03 (13.31)
DASS-Depression	8.69 (5.12)	9.53 (5.07)	9.42 (5.73)	9.15 (5.28)
DASS-Anxiety	8.52 (5.80)	8.95 (5.51)	8.78 (5.84)	8.72 (5.71)
ASI-3	24.56 (14.54)	26.35 (13.50)	23.77 (13.67)	24.88 (13.96)
Weekly Mins ModVig PA	210.65 (191.64)	109.76 (176.82)	117.94 (154.39)	154.45 (183.12)
Weekly Mins Mild PA	158.17 (147.44)	134.49 (162.93)	168.05 (210.35)	153.69 (171.47)

*Note*. ASI-3: Anxiety Sensitivity Index – 3; DASS: Depression Anxiety Stress Scale – 21; Mins: minutes; ModVig: moderate-to-vigorous; PA: physical activity; Psych DX: ever been diagnosed with a psychological disorder (yes/no).

### Materials

#### Demographics

Participants’ age, sex, marital status, and income was collected. For those with a PD, information about the nature of the impairment was collected (e.g. diagnosis, whether it is congenital or acquired, whether mobility aids are needed).

#### Anxiety sensitivity

AS was assessed using the 18-item, self-report Anxiety Sensitivity Index – 3 (ASI-3; Taylor et al., 2007). Participants indicate how strongly they agree (*0* = *very little* to *4* = *very much*) with statements (e.g. “It scares me when my heart beats rapidly”) that describe experiencing fear in response to physiological sensations. Item scores are summed to get a total score. The internal reliability of the ASI-3 was excellent in the current study (*α* = 0.92). In this sample, AS and anxiety had a significant medium correlation (*r* = 0.41, *p* < 0.001) similar to the correlation between AS and depressive symptoms (*r* = 0.39, *p* < 0.001).

#### Anxiety and depression

Symptoms of anxiety and depression were measured using the Depression Anxiety Stress Scale - 21 (DASS-21; Lovibond and Lovibond, 1995). The DASS-21 is composed of three subscales: depression, anxiety, and stress. Individuals indicate the extent to which a particular negative emotional state (e.g. “I found it difficult to relax”) applied to them over the past week (*0* = *Did not apply to me at all* to *3* = *Applied to me very much or most of the time*). Scores are obtained by summing items pertaining to each subscale. For this study, only the anxiety and depression subscales were used. We selected the DASS-21 because items do not reference the somatic symptoms of depression. This is important because many people with PDs will rate somatic symptoms highly, giving falsely high ratings of depression (Müller et al., 2012). The DASS-21 has been used in multiple studies with people with a PD (Mitchell et al., 2008). Reliability for the current sample was adequate for the depression (*α* = 0.70) and anxiety (*α* = 0.73) subscales.

#### Physical activity

Physical activity was measured using the International Godin-Shephard Leisure-Time Physical Activity Questionnaire (GSLTPAQ; Godin, 2011). This measure asks participants how often they do mild, moderate, and vigorous intensity leisure-time PA (at least 15-minute intervals) in a typical week. In line with prior research, the proffered examples of mild, moderate, and vigorous intensity PA were modified to reflect activities that may be performed by individuals with a PD (Bombardier et al., 2012). Additionally, an intensity chart from the Physical Activity Recall Assessment for People with a Spinal Cord Injury (Martin Ginis et al., 2005) was provided to ensure every participant used the same reference material when identifying intensity. For this study (as in previous research; Jung and Brawley, 2013), three questions were added: “On average how many minutes do you engage in [vigorous/moderate/mild leisure-time] physical activity at a time?” This way, multiplying the number of bouts by their typical length generates three scores representing minutes of mild, moderate, and vigorous intensity PA per week. The GSLTPAQ has been shown to be a reliable and appropriate tool for individuals with a variety of PDs (Sikes et al., 2019).

### Procedure

Participants were initially recruited through online advertisements placed on social media groups related to disability (e.g. Cerebral Palsy Positive, Paralyzed Veterans of America, National Center on Health, Physical Activity, and Disability). Advertisements contained a link to the study’s self-report survey hosted on Qualtrics^®^, a confidential and secure online survey platform. Following challenges with recruitment, the study was posted on Prolific (an online research platform) to recruit the remaining participants. An a priori power analysis using G*Power revealed that 191 participants (64 per disability group) would be needed to achieve sufficient power for the planned analyses. All participants completed an online consent form before being directed to the study survey, which took participants approximately 20 minutes to complete.

### Data analysis

To test our hypotheses moderation analyses (Tables 2 and 3) were conducted using the Process Macro for SPSS (Hayes, 2022). Moderators were tested in the associations between (1) moderate-to-vigorous PA and depression, (2) mild PA and depression, (3) moderate-to-vigorous PA and anxiety, and (4) mild PA and depression. Age and gender were entered as covariates as they are known to be associated with anxiety and depression and PA (Azevedo et al., 2007) and given group differences in age in the current sample. For all models, we first used Helmert contrasts to test whether the disability group (DIS; acquired and congenital combined) differed from the no disability group (NO) and whether the acquired disability group (ACQ) differed from the congenital disability group (CON). When Helmert contrasts suggested significant moderation, follow-up moderation analyses were conducted using Effect contrasts to determine if the no disability group was different from the acquired disability group (NO vs ACQ) and/or the congenital disability group (NO vs CON).

**Table 2.** Moderating role of disability status in the association between PA and depression.

Predictor variables	ModVig PA Predicting depression	Mild PA Predicting depression
	*b* (95% CI)	*b* (95% CI)
Model	*F*(7, 217) = 6.80***	*F*(7, 219) = 4.28***
Gender	0.65 (−0.48 to 1.77)	0.12 (−1.02 to 1.25)
Age	−0.14*** (−0.19 to −0.09)	−0.13*** (−0.18 to −0.08)
PA	−0.005* (−0.01 to −0.001)	−0.0003 (−0.004 to 0.004)
DIS vs NO	1.28 (−0.10 to 2.66)	1.55* (0.18 to 2.93)
ACQ vs CON	−1.38 (−3.13 to 0.37)	−0.85 (−2.59 to 0.89)
NO vs ACQ	1.12* (0.13 to 2.10)	--------
NO vs CON	−0.26 (−1.26 to 0.73)	--------
DIS vs NO × PA	−0.002 (−0.01 to 0.01)	0.003 (−0.01 to 0.01)
ACQ vs CON × PA	−0.02*** (−0.03 to −0.01)	−0.004 (−0.01 to 0.01)
NO vs ACQ × PA	0.01** (0.003 to 0.01)	-------
NO vs CON × PA	−0.01** (−0.02 to −0.004)	-------

*Note*. ACQ: acquired disability; CON: congenital disability; DIS: disability; NO: no disability; ModVig: moderate-to-vigorous; PA: physical activity.

* *p* < 0.05, **p < 0.01, ***p < 0.001.

**Table 3.** Moderating role of disability status in the association between PA and anxiety.

Predictor variables	ModVig PA Predicting anxiety	Mild PA Predicting anxiety
	*b* (95% CI)	*b* (95% CI)
Model	*F*(7, 217) = 3.71***	*F*(7, 219) = 3.07**
Gender	0.69 (-0.58 to 1.96)	0.31 (−0.94 to 1.57)
Age	−0.12*** (−0.18 to −0.07)	−0.12*** (−0.17 to -0.06)
PA	−0.003 (−0.01 to 0.001)	0.001 (−0.003 to 0.01)
DIS vs. NO	0.93 (−0.62 to 2.49)	1.12 (−0.40 to 2.64)
ACQ vs. CON	−1.07 (−3.05 to 0.91)	−0.83 (−2.76 to 1.11)
NO vs. ACQ	0.84 (-0.27 to 1.96)	--------
NO vs. CON	−0.22 (−1.34 to 0.90)	--------
DIS vs. NO × PA	0.001 (−0.01 to 0.01)	0.01 (−0.002 to 0.02)
ACQ vs. CON × PA	−0.01* (-0.03 to -0.001)	−0.002 (−0.01 to 0.01)
NO vs. ACQ × PA	0.01* (0.001 to 0.01)	-------
NO vs. CON × PA	−0.01 (−0.01 to 0.001)	-------

*Note*. ACQ: acquired disability; CON: congenital disability; DIS: disability; NO: no disability; ModVig: moderate-to-vigorous; PA: physical activity.

* *p* < 0.05, **p < 0.01, ***p < 0.001.

Next, to test the moderating role of disability status in the relation between PA and AS, we followed the same data analytic approach described above with AS as the outcome. Given the lack of association between mild PA and anxiety or depression, we conducted these analyses using moderate-to-vigorous PA. Next, we tested the mediating role of AS on the association between PA and anxiety and depression and if this mediation was moderated by disability status. The model tested for moderation at both the *a* (PA predicting AS) and *c’* (PA predicting anxiety and depression) pathways as the previous analyses showed moderation in both pathways. As with previous analyses, age and gender were entered as covariates. Given the lack of association between mild PA and anxiety or depression, moderated mediation was tested with moderate-to-vigorous PA.

## Results

### Data screening

About 334 participants consented and completed the study questionnaire. Seventy-eight participants failed an attention check and were removed. Three attention check questions were included in the questionnaire where participants were asked to answer in a specific way (e.g. “Please select *strongly disagree for this* statement”); if they did not, this was considered a failed attention check. A missing values analysis found that <5% of the data was missing. Case mean substitution was used to input missing values for those who completed 80% of a given scale. Those completing <80% of any measure were excluded from analyses using those measures. *Z*-scores were obtained and 19 univariate outliers were identified and deleted. Mahalanobis distances were used to determine multivariate outliers and 6 multivariate outliers were identified and deleted. This left a sample of 231.

Almost half (44.6%) of participants reported 0 minutes of vigorous PA, making this variable highly skewed. As such, the vigorous and moderate PA variables were combined. This has been done frequently in past research as both intensities are often found to be associated with mental health (Loprinzi, 2013; Nakagawa et al., 2020).

One-way analyses of variance (ANOVAs) were used to test for differences on variables between those with a congenital, acquired, or no disability. There were only significant between-group differences in age, *F* = 7.37, *p* < 0.001, and moderate-to-vigorous PA, *F* = 8.25, *p* < 0.001. Because age violated assumptions of homogeneity of variance, the Games-Howell post-hoc test was used to explore between-group differences. For moderate-to-vigorous PA, Tukey’s post-hoc test was used. Post-hoc comparisons revealed that those with no disability were significantly younger than those with acquired disabilities and reported significantly more moderate-to-vigorous PA than those with an acquired or congenital disability.

### Moderating role of disability in the associations between physical activity and anxiety and depression

**Figure 1.**
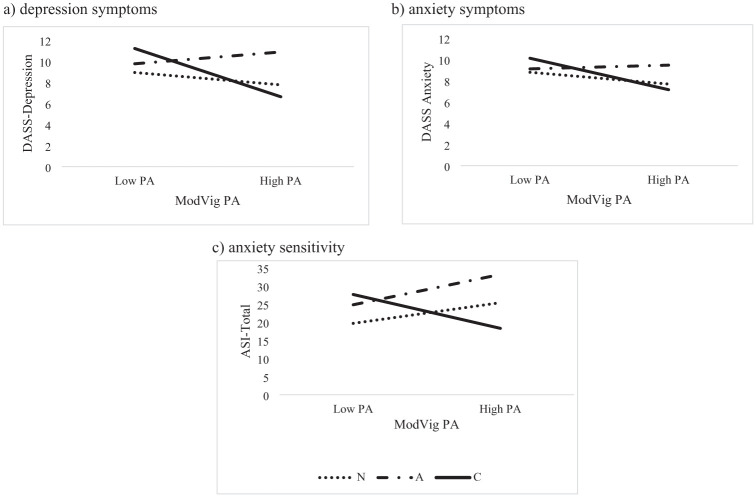
The moderating role of disability status in the association between moderate-to-vigorous PA and mental health: (a) depression symptoms, (b) anxiety symptoms, and (c) anxiety sensitivity. *Note*. A: acquired disability; C: congenital disability; N: no disability; ASI: Anxiety Sensitivity Index – 3; DASS-Anxiety: Depression Anxiety Stress Scales – 21 Anxiety subscale; DASS-Depression: Depression Anxiety Stress Scales – 21 Depression subscale; ModVig PA: moderate-to-vigorous physical activity.

When moderate-to vigorous PA was the predictor and depression the outcome, Helmert contrasts revealed significant moderations by disability status (DIS vs NO and ACQ vs CON). Follow-up Effect contrasts revealed significant moderations when comparing the NO versus ACQ and NO versus CON groups. When looking at the conditional effects, there was no significant association between moderate-to-vigorous PA and depression for the NO group (*b* = −0.004) or the ACQ group (*b* = 0.004), but for the CON group more moderate-to-vigorous PA was significantly associated with less depressive symptoms (*b* = −0.02; Figure 1). When mild PA was the predictor and depression was the outcome, Helmert contrasts showed that disability status was not a moderator.

When moderate-to-vigorous PA was the predictor and anxiety was the outcome, Helmert contrasts found a significant moderation when comparing the ACQ versus CON groups. Follow-up Effect contrasts showed a significant interaction when comparing the NO versus ACQ groups. Looking at the conditional effects, there was no significant association between moderate-to-vigorous PA and anxiety for the NO (*b* = −0.004) and ACQ (*b* = 0.004) groups, but for those in the CON group (*b* = −0.01, *p* = 0.044), more moderate-to-vigorous PA was significantly associated with less anxiety symptoms (*b* = −0.01; Figure 1). When mild PA was the predictor and anxiety the outcome, disability status was not a moderator according to Helmert contrasts.

### Moderating role of disability status in the relation between physical activity and anxiety sensitivity

Helmert contrasts showed that disability status moderated the association between moderate-to-vigorous PA and AS (DIS vs NO and ACQ vs CON). Effect contrasts also showed significant moderation when comparing the NO versus ACQ groups and the NO versus CON groups. When looking at the conditional effects, the effect of moderate-to-vigorous PA on AS was significant and positive for the NO (*b* = 0.02), and ACQ (*b* = 0.03) groups but significant and negative for the CON group (*b* = −0.03; Figure 1; Table 4). In other words, for those with no or an acquired disability, more moderate-to-vigorous PA was associated with higher AS while for those with congenital disabilities, more moderate-to-vigorous PA was associated with lower AS.

**Table 4.** Association between PA and AS with disability status as a moderator.

Predictor variables	ModVig PA b (95% C.I)
Model	*F*(7, 217) = 6.49***
Gender	2.40 (−0.58 to 5.39)
Age	−0.29*** (−0.42 to −0.15)
PA	0.01 (−0.01 to 0.02)
DIS vs NO	3.47 (−0.17 to 7.12)
ACQ vs CON	−5.95* (−10.59 to −1.31)
NO vs ACQ	4.13** (1.52 to 6.75)
NO vs CON	−1.82 (−4.45 to 0.81)
DIS vs NO × PA	−0.02* (−0.04 to −0.0003)
ACQ vs CON × PA	−0.06*** (−0.08 to −0.03)
NO vs ACQ × PA	0.02** (0.01 to 0.04)
NO vs CON × PA	−0.03*** −0.05 to −0.02)

*Note*. ACQ: acquired disability; AS: anxiety sensitivity; CON: congenital disability; DIS: disability; NO: no disability; ModVig: moderate-to-vigorous; PA: physical activity.

* *p* < 0.05, **p < 0.01, ***p < 0.001.

### Moderated mediation

First, we tested the model with depression as the outcome. Helmert contrasts resulted in a significant index of moderated mediation when comparing the NO versus DIS (*index* = −0.002, *95% C.I.* = −0.01 to −0.0001) and ACQ versus CON (*index* = −0.01, *95%C.I.* = −0.01 to −0.004) groups. Effect contrasts revealed the index of moderated mediation was significant when comparing the NO versus ACQ (*index* = −0.003, *95% C.I.* = 0.001 to 0.005), and NO versus CON (*index* = −0.004, *95%C.I.* = −0.01 to −0.002) groups. The indirect effect of AS on the relation between moderate-to-vigorous PA and depression was positive and significant for the NO (*b* = 0.002, *95% C.I.* = 0.001 to 0.005) and ACQ (*b* = 0.003, *95% C.I.* = 0.002 to 0.01) groups, and negative and significant for the CON group (*b* = −0.004, *95% C.I.* = −0.01 to −0.002). In other words, for those with no disabilities and acquired disabilities, the positive association between moderate-to-vigorous PA and depression was partially mediated by AS while for those with congenital disabilities, the negative association between moderate-to-vigorous PA and depression was partially mediated by AS.

Next, we tested the model with anxiety as the outcome. Helmert contrasts indicated that moderated mediation was present when comparing the NO versus DIS (*index* = -0.003, *95%C.I.* = −0.01 to −0.0002) and ACQ versus CON (*index* = −0.01, *95% C.I.* = −0.01 to −0.005) groups. Effect contrasts indicated that moderated mediation was present when comparing the NO versus ACQ (*index* = 0.003, *95%C.I.* = 0.002 to 0.01) and NO versus CON (*index* = −0.01, *95%C.I.* = −0.01 to −0.003) groups. The indirect effect of AS on the association between moderate-to-vigorous PA and anxiety was positive and significant for those in the NO (*b* = 0.003, *95%C.I.* = 0.001 to 0.01) and ACQ (*b* = 0.004, *95%C.I.* = 0.002 to 0.01) groups, but negative and significant for the CON group (*b* = −0.005, *95%C.I.* = −0.01 to −0.002). These findings mirrored the findings emerging from the model with depression.

## Discussion

This study aimed to better understand the association between PA and anxiety and depression in those with and without PDs. Although previous research has suggested that PA is associated with mental health outcomes for individuals with and without disabilities, few direct comparisons exist testing differences in this relation (Battalio et al., 2020; Rosenberg et al., 2013; Zeibig et al., 2021). Results revealed several significant moderation pathways, suggesting that the associations between PA and both anxiety and depression symptoms differ according to disability status. Since the pattern of results did not change meaningfully for anxiety and depressive symptoms, results will be discussed together.

As hypothesized, the association between moderate-to-vigorous PA and anxiety/depressive symptoms was moderated by disability status. Specifically, there was no association between moderate-to-vigorous PA and anxiety/depressive symptoms for those with no disability or an acquired disability while PA and anxiety/depression were significantly, negatively associated for those with a congenital disability (Ehlers Danlos syndrome, MD, cerebral palsy, etc.). Disability status did not, however, moderate the association between mild PA and anxiety/depressive symptoms. Furthermore, there was no association between mild PA and anxiety/depressive symptoms regardless of disability status. These findings align with much of the prior literature that has found that higher (vs lower) intensity PA is associated with anxiety and depression symptoms (Battalio et al., 2020; Zeibig et al., 2021). Nevertheless, some studies have found that mild PA may be significantly associated with anxiety and depression in a SCI sample (Connell and Olthuis, 2023; Tawashy et al., 2009). This suggest that there may be a role for lower intensity PA in the reduction of anxiety and depressive symptoms in certain disability groups, however, more research is needed to better understand these nuances.

When it comes to moderate-to-vigorous PA for those with an acquired disability, the change in how one experiences PA after a disability may affect how PA relates to psychological concepts such as anxiety and depression (Connell and Olthuis, 2023). Specifically, the physical sensations experienced during PA may change, the difficulty of PA may increase, and PA may highlight an individual’s new physical limitations. Indeed, qualitative research participants have reported that “. . .It [PA] doesn’t feel good. . .at all. . . I don’t get sweaty. . . I don’t get my heart rate up” (Kehn and Kroll, 2009, p. 5). Others reported that the benefits of PA did not outweigh the time and effort required to participate, or that PA was frustrating and disappointing (Kehn and Kroll, 2009). We might expect then that PA is not negatively associated with anxiety and depression in those with acquired disabilities without intervention (Rebar et al., 2015; Weinstein et al., 2017; Wipfli et al., 2008), thus explaining the lack of association between PA and anxiety/depression in this study. Other studies have also failed to find significant associations between moderate-to-vigorous PA and anxiety and depression for those with an acquired disability (Bombardier et al., 2012; Connell and Olthuis, 2023; Tawashy et al., 2009).

In contrast, there was a significant, negative association between moderate-to-vigorous PA and depression/anxiety for those with a congenital disability. This is consistent with prior research among those with neuromuscular disorders (Vita et al., 2020). Because those with congenital disabilities have likely experienced symptoms associated with their disability since birth, they may be habituated to unpleasant sensations or symptoms (e.g. pain, numbness, tingling), may be more used to their physical limitations, and may be able to find more joy in PA. Thus, PA may reduce depression/anxiety for those with a congenital disability in a similar fashion as it has done in prior studies with the non-disability samples (Rebar et al., 2015; Weinstein et al., 2017; Wipfli et al., 2008).

Notably, however, in the current study there was no association between PA and anxiety/depression in those with no disability. Given prior research (Rebar et al., 2015; Weinstein et al., 2017; Wipfli et al., 2008), we had expected to find a significant, negative association. This lack of association may be due to the high AS nature of the sample (the mean ASI-3 score was >1 *SD* above the typical population mean; Reiss et al., 2008). Research has shown that the amount of enjoyment one feels during PA affects its association with mental health (Abrantes et al., 2017). Those with high (vs low) AS tend to experience more distress during PA (Moshier et al., 2016; Sabourin et al., 2011; Smits et al., 2010). Thus, it is possible that many participants in the current study do not enjoy PA, contributing to its lack of association with anxiety/depression in those without a disability.

To better understand the associations between PA and anxiety/depression by disability status, we investigated the possible role of AS in these associations. As hypothesized, the association between PA and AS was moderated by disability status. The directions of the associations, however, were not as hypothesized. We expected a negative association between PA and AS for those with no disability (in line with prior research; DeWolfe et al., 2023) and a weaker association between PA and AS for those with an acquired or congenital disability (also in line with prior research; Connell and Olthuis, 2023). Instead, we found a significant, positive association between PA and AS for those with no disability and an acquired disability and a significant, negative association between PA and AS for those with a congenital disability. While this finding evidently shows that disability status matters in the association between PA and AS, it raises more questions about the nature of these associations.

Although the positive association between PA and AS in those with no disability and an acquired disability seems to contradict prior research, a recent menta-analysis (DeWolfe et al., 2023) found that in those with clinical levels of anxiety and depression, the association between PA and AS weakens. The authors hypothesized that this could be due to several factors that frequently co-occur in individuals with high anxiety and depression, such as high levels of health anxiety, which have been associated with increased PA (Lees et al., 2005). In the current sample, levels of depression were moderate, and levels of anxiety were severe (Lovibond and Lovibond, 1995). It may be that this sample characteristic contributed to the positive association between PA and AS among those without a disability and those with an acquired disability. Though we did not measure health anxiety in the current study, prior research has shown that individuals with MS (an acquired disability) have higher health anxiety than age-matched controls (Kehler and Hadjistavropoulos, 2009).

Unlike those with no disability or an acquired disability, the association between moderate-to-vigorous PA and AS was significant and negative for those with a congenital disability. These results align with prior research with non-disabled populations showing that PA can reduce AS over time (Jacquart et al., 2019). It may be that despite the unique physiological functioning experienced by those with a congenital disability, PA still exposes and habituates them to a range of arousal-related sensations.

Finally, moderated mediation models revealed a mediating role for AS in the association between moderate-to-vigorous PA and anxiety/depression regardless of disability status. This means that part of the association between moderate-to-vigorous PA and anxiety and depression is explained by AS. However, the magnitude and direction of the mediating role of AS differed among the no disability, acquired disability, and congenital disability groups. These findings are consistent with the previous moderation analyses showing a significant, positive role of AS in the relation between moderate-to-vigorous PA and anxiety/depression for those in the no disability group and acquired disability group and a negative role of AS in this association for those in the congenital disability group. These moderated mediation findings highlight the role of AS in the association between PA and anxiety and depression, even in those with a PD.

### Limitations

This study should be considered in light of several limitations. First, it is cross-sectional, meaning causation cannot be determined. The mediation models should be interpreted cautiously for this same reason. Second, the use of a self-report questionnaire creates opportunity for social desirability bias. This is especially true for measures of PA (Prince et al., 2008). Future research should also focus on longitudinal studies that use more objective measures of PA, such as wearable PA trackers. Finally, due to the structure of the analyses, time since diagnosis (for the acquired disability group) could not be included in analyses, which may be significantly associated with anxiety and depressive symptoms.

## Conclusion

Taken together, this study shows an important role for disability status, AS, and their interaction, in explaining the association between PA and anxiety and depression. While the direction of some of the associations in this study were surprising, results clearly suggest that the association between PA and anxiety and depression is influenced by the experience of physiological sensations (i.e. AS) which appears to be affected by PD. While mental health status of the sample may explain some of the unexpected associations, future studies (that consider mental health status) are needed to replicate the existing findings. Importantly, this study illustrates the potential nuances that are lost when those with PDs are treated as one group in research. Although there may be similarities in the symptoms experienced by those with PDs, there are clearly differences in lived experiences amongst these diverse groups of individuals. Although the association between PA and mental health is complex and multi-faceted, and it is impossible to account for all the factors that may affect this association, future studies should be mindful when grouping individuals with disabilities together.
